# Hexa-μ-chlorido-μ_4_-oxido-tetra­kis­({1-[(pyridin-2-yl)meth­yl]-1*H*-benzimidazole-κ*N* ^3^}copper(II))

**DOI:** 10.1107/S1600536811035252

**Published:** 2011-09-14

**Authors:** Hui Li, Hongshi Jiang, Hong Sun

**Affiliations:** a Department of Applied Chemistry, Yuncheng University, Yuncheng, Shanxi 044000, People’s Republic of China

## Abstract

The title tetra­nuclear complex, [Cu_4_Cl_6_O(C_13_H_11_N_3_)_4_], features a tetra­hedral arrangement of copper(II) ions bonded to the central O atom (site symmetry 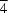). Each of the six edges of the Cu_4_ tetra­hedron is bridged by a chloride ion (one of which has site symmetry 2), so that each copper ion is linked to the other three metal ions through the central O atom and through three separate chloride-ion bridges. The fifth coord­ination position, located on the central Cu—O axis on the outside of the cluster, is occupied by an N atom of the mono­dentate 1-(pyridin-2-ylmeth­yl)-1*H*-benzimidazole ligand. The resulting coordination geometry of the metal ion is a distorted trigonal bipyramid with the O and N atoms in the axial positions. The dihedral angle between the benzimidazole ring system and the pendant pyridine ring is 61.0 (2)°.

## Related literature

For background to polynuclear copper halides, see: Willett (1991 ▶); Chivers *et al. *(2005 ▶); Li *et al.* (2009 ▶). 
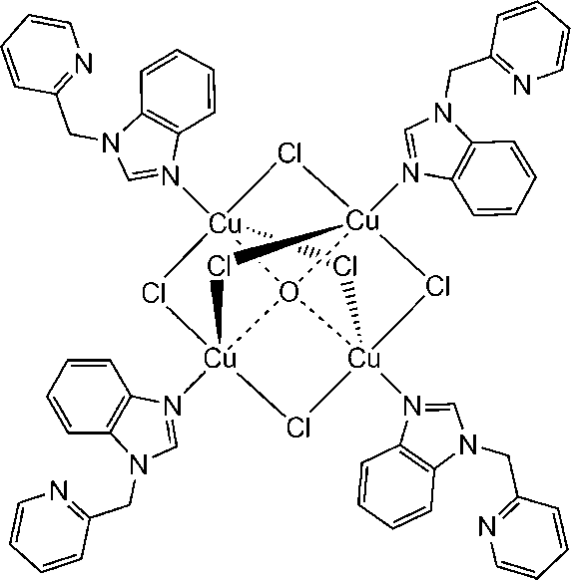


## Experimental

### 

#### Crystal data

[Cu_4_Cl_6_O(C_13_H_11_N_3_)_4_]
*M* *_r_* = 1319.85
Tetragonal, 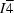

*a* = 13.8532 (12) Å
*c* = 14.507 (3) Å
*V* = 2784.1 (6) Å^3^
*Z* = 2
Mo *K*α radiation
μ = 1.85 mm^−1^
*T* = 294 K
0.25 × 0.23 × 0.20 mm

#### Data collection

Rigaku Mercury CCD diffractometer
Absorption correction: multi-scan (*CrystalClear*; Rigaku/MSC, 2005 ▶) *T*_min_ = 0.637, *T*_max_ = 0.691
7149 measured reflections
2467 independent reflections
2178 reflections with *I* > 2σ(*I*)
*R*_int_ = 0.033

#### Refinement

*R*[*F* ^2^ > 2σ(*F* ^2^)] = 0.028
*wR*(*F* ^2^) = 0.062
*S* = 1.06
2467 reflections
170 parameters
H-atom parameters constrained
Δρ_max_ = 0.34 e Å^−3^
Δρ_min_ = −0.17 e Å^−3^
Absolute structure: Flack (1983) ▶, 1172 Friedel pairs
Flack parameter: 0.005 (15)

### 

Data collection: *CrystalClear* (Rigaku/MSC, 2005 ▶); cell refinement: *CrystalClear*; data reduction: *CrystalClear*; program(s) used to solve structure: *SHELXS97* (Sheldrick, 2008 ▶); program(s) used to refine structure: *SHELXL97* (Sheldrick, 2008 ▶); molecular graphics: *SHELXTL* (Sheldrick, 2008 ▶); software used to prepare material for publication: *SHELXTL*.

## Supplementary Material

Crystal structure: contains datablock(s) I, global. DOI: 10.1107/S1600536811035252/hb6384sup1.cif

Structure factors: contains datablock(s) I. DOI: 10.1107/S1600536811035252/hb6384Isup2.hkl

Additional supplementary materials:  crystallographic information; 3D view; checkCIF report

## Figures and Tables

**Table 1:** Selected bond lengths (Å)

Cu1—O1	1.9199 (4)
Cu1—N3	1.974 (3)
Cu1—Cl1^i^	2.3961 (10)
Cu1—Cl1	2.4192 (10)
Cu1—Cl2	2.4263 (10)

Symmetry code: (i) 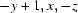.

**Fig. 1.**
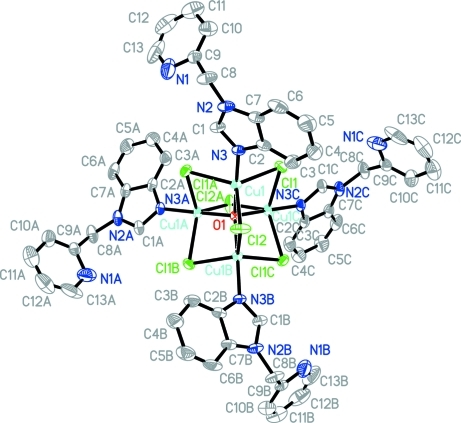
The molecular structure of (I). Displacement ellipsoids are drawn at the 30% probability level and H atoms are removed for clarity. [symmetry code: (A) -y + 1, x, -z; (B) y, -x + 1, -z; (C) -x + 1, -y + 1, z].

**Fig. 2.**
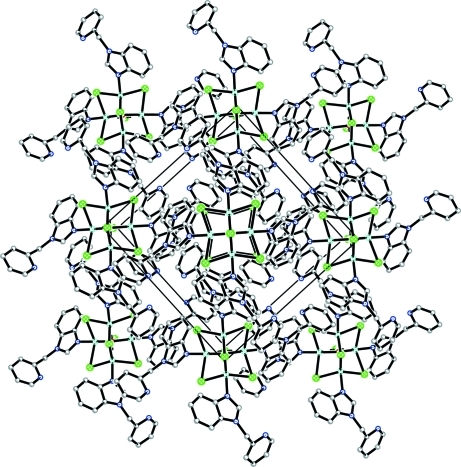
The crystal packing for (I) viewed along the c axis.

## supplementary crystallographic information

### Comment

Copper(II) halide framework materials have attracted much attention for their interesting magnetic properties and structural richness (Willett *et al.*, 1991). The most commonly employed technique to modulate the inorganic network involves the direct addition of an organic ligand as a templating reagent (Chivers *et al.*, 2005). benzimidazole has been well used in crystal engineering, and a large number of benzimidazole ligands have been extensively studied (Li *et al.*, 2009). The reaction of CuCl_2_ with the benzimidazole-pyridine ligand (L) affords a tetranuclear molecule [(Cu_4_O)Cl_6_(L)_4_], (I). The crystal structure was elucidated by X-ray diffraction analysis.

### Experimental

To a solution of L (0.12 mmol, 25 mg) dissolved in CH_3_CN (9 ml), a solution of CuCl_2_^.^6H_2_O (0.12 mmol, 28.9 mg) in H_2_O (9 ml) was added under stirring in a few minutes. The solution was left to stand at room temperature. Brown blocks of (I) were obtained after several days with solvent evaporation. Yield: ~20% (based on L).

### Refinement

C-bound H atoms were positioned geometrically and refined in the riding-model approximation, with C—H = 0.93Å and *U*_iso_(H) = 1.2Ueq.

### Crystal data

**Table:**

[Cu_4_Cl_6_O(C_13_H_11_N_3_)_4_]	*D*_x_ = 1.574 Mg m^−^^3^
*M**_r_* = 1319.85	Mo *K*α radiation, λ = 0.71073 Å
Tetragonal, *I*4	Cell parameters from 3492 reflections
*a* = 13.8532 (12) Å	θ = 2.8–25.3°
*c* = 14.507 (3) Å	µ = 1.85 mm^−^^1^
*V* = 2784.1 (6) Å^3^	*T* = 294 K
*Z* = 2	Block, brown
*F*(000) = 1332	0.25 × 0.23 × 0.20 mm

### Data collection

**Table:**

Rigaku Mercury CCD diffractometer	2467 independent reflections
Radiation source: fine-focus sealed tube	2178 reflections with *I* > 2σ(*I*)
graphite	*R*_int_ = 0.033
Detector resolution: 9 pixels mm^-1^	θ_max_ = 25.0°, θ_min_ = 2.0°
ω scans	*h* = −14→16
Absorption correction: multi-scan (*CrystalClear*; Rigaku/MSC, 2005)	*k* = −16→14
*T*_min_ = 0.637, *T*_max_ = 0.691	*l* = −17→17
7149 measured reflections	

### Refinement

**Table:**

Refinement on *F*^2^	Secondary atom site location: difference Fourier map
Least-squares matrix: full	Hydrogen site location: inferred from neighbouring sites
*R*[*F*^2^ > 2σ(*F*^2^)] = 0.028	H-atom parameters constrained
*wR*(*F*^2^) = 0.062	*w* = 1/[σ^2^(*F*_o_^2^) + (0.0286*P*)^2^] where *P* = (*F*_o_^2^ + 2*F*_c_^2^)/3
*S* = 1.06	(Δ/σ)_max_ = 0.001
2467 reflections	Δρ_max_ = 0.34 e Å^−^^3^
170 parameters	Δρ_min_ = −0.17 e Å^−^^3^
0 restraints	Absolute structure: Flack (1983), 1172 Friedel pairs
0 constraints	Flack parameter: 0.005 (15)
Primary atom site location: structure-invariant direct methods	

### Special details

**Table:**

Geometry. All e.s.d.'s (except the e.s.d. in the dihedral angle between two l.s. planes) are estimated using the full covariance matrix. The cell e.s.d.'s are taken into account individually in the estimation of e.s.d.'s in distances, angles and torsion angles; correlations between e.s.d.'s in cell parameters are only used when they are defined by crystal symmetry. An approximate (isotropic) treatment of cell e.s.d.'s is used for estimating e.s.d.'s involving l.s. planes.
Refinement. Refinement of *F*^2^ against ALL reflections. The weighted *R*-factor *wR* and goodness of fit *S* are based on *F*^2^, conventional *R*-factors *R* are based on *F*, with *F* set to zero for negative *F*^2^. The threshold expression of *F*^2^ > σ(*F*^2^) is used only for calculating *R*-factors(gt) *etc*. and is not relevant to the choice of reflections for refinement. *R*-factors based on *F*^2^ are statistically about twice as large as those based on *F*, and *R*- factors based on ALL data will be even larger.

### Fractional atomic coordinates and isotropic or equivalent isotropic displacement parameters (Å^2^)

**Table:**

	*x*	*y*	*z*	*U*_iso_*/*U*_eq_	
Cu1	0.42929 (3)	0.41004 (3)	0.07467 (3)	0.03400 (12)	
Cl1	0.28847 (6)	0.47255 (6)	−0.00384 (7)	0.0473 (2)	
Cl2	0.5000	0.5000	0.20129 (8)	0.0637 (4)	
N3	0.3550 (2)	0.3189 (2)	0.1515 (2)	0.0412 (7)	
C1	0.3592 (3)	0.2243 (3)	0.1520 (3)	0.0498 (10)	
H1	0.3992	0.1887	0.1133	0.060*	
C2	0.2867 (3)	0.3436 (3)	0.2180 (2)	0.0485 (10)	
C7	0.2518 (3)	0.2588 (3)	0.2595 (3)	0.0533 (10)	
N2	0.2998 (3)	0.1839 (2)	0.2144 (2)	0.0548 (9)	
C6	0.1844 (3)	0.2627 (4)	0.3322 (3)	0.0736 (15)	
H6	0.1636	0.2068	0.3616	0.088*	
C3	0.2513 (3)	0.4325 (3)	0.2461 (3)	0.0663 (13)	
H3	0.2726	0.4892	0.2184	0.080*	
C4	0.1837 (4)	0.4350 (4)	0.3161 (4)	0.0844 (16)	
H4	0.1593	0.4940	0.3359	0.101*	
C5	0.1515 (4)	0.3495 (5)	0.3574 (4)	0.0899 (18)	
H5	0.1056	0.3532	0.4040	0.108*	
C8	0.2900 (4)	0.0806 (3)	0.2368 (3)	0.0755 (15)	
H8A	0.2343	0.0721	0.2764	0.091*	
H8B	0.3465	0.0601	0.2711	0.091*	
O1	0.5000	0.5000	0.0000	0.0292 (9)	
C9	0.2789 (3)	0.0170 (3)	0.1543 (3)	0.0563 (10)	
C10	0.1944 (4)	−0.0195 (4)	0.1286 (4)	0.0846 (16)	
H10	0.1380	−0.0038	0.1600	0.102*	
N1	0.3632 (4)	−0.0017 (4)	0.1100 (4)	0.1075 (18)	
C12	0.2815 (9)	−0.1018 (5)	0.0115 (5)	0.128 (3)	
H12	0.2863	−0.1449	−0.0374	0.154*	
C11	0.1926 (7)	−0.0858 (5)	0.0487 (5)	0.116 (2)	
H11	0.1364	−0.1138	0.0261	0.139*	
C13	0.3600 (8)	−0.0601 (5)	0.0405 (6)	0.140 (4)	
H13	0.4172	−0.0730	0.0093	0.168*	

### Atomic displacement parameters (Å^2^)

**Table:**

	*U*^11^	*U*^22^	*U*^33^	*U*^12^	*U*^13^	*U*^23^
Cu1	0.0374 (2)	0.0322 (2)	0.03237 (19)	−0.00702 (17)	0.0024 (2)	0.00311 (19)
Cl1	0.0319 (5)	0.0549 (5)	0.0552 (5)	−0.0045 (4)	−0.0026 (4)	0.0094 (5)
Cl2	0.0911 (11)	0.0707 (10)	0.0294 (6)	−0.0472 (9)	0.000	0.000
N3	0.0419 (18)	0.0393 (18)	0.0425 (16)	−0.0130 (14)	0.0009 (14)	0.0065 (14)
C1	0.061 (3)	0.045 (2)	0.043 (2)	−0.0162 (19)	−0.006 (2)	0.0044 (19)
C2	0.045 (2)	0.058 (3)	0.042 (2)	−0.0166 (19)	−0.0002 (18)	0.007 (2)
C7	0.054 (2)	0.060 (3)	0.045 (2)	−0.021 (2)	0.001 (2)	0.010 (2)
N2	0.068 (2)	0.048 (2)	0.048 (2)	−0.0231 (17)	−0.0028 (18)	0.0152 (17)
C6	0.064 (3)	0.096 (4)	0.061 (3)	−0.035 (3)	0.005 (2)	0.030 (3)
C3	0.073 (3)	0.060 (3)	0.066 (3)	−0.015 (2)	0.021 (3)	0.003 (2)
C4	0.083 (4)	0.080 (4)	0.091 (4)	−0.003 (3)	0.037 (3)	0.002 (3)
C5	0.084 (4)	0.102 (5)	0.084 (4)	−0.016 (3)	0.040 (3)	0.002 (3)
C8	0.108 (4)	0.054 (3)	0.065 (3)	−0.030 (3)	−0.008 (3)	0.021 (2)
O1	0.0293 (14)	0.0293 (14)	0.029 (2)	0.000	0.000	0.000
C9	0.064 (3)	0.042 (2)	0.064 (3)	−0.002 (2)	0.010 (2)	0.020 (2)
C10	0.093 (4)	0.081 (4)	0.081 (4)	−0.020 (3)	0.010 (3)	0.001 (3)
N1	0.107 (4)	0.085 (3)	0.130 (5)	0.035 (3)	0.033 (3)	0.025 (3)
C12	0.229 (11)	0.066 (4)	0.090 (5)	−0.007 (6)	0.033 (7)	0.000 (4)
C11	0.158 (7)	0.092 (5)	0.097 (5)	−0.030 (5)	−0.005 (5)	0.008 (4)
C13	0.202 (10)	0.064 (5)	0.154 (8)	0.052 (5)	0.079 (7)	0.012 (5)

### Geometric parameters (Å, °)

**Table:**

Cu1—O1	1.9199 (4)	C4—C5	1.400 (7)
Cu1—N3	1.974 (3)	C4—H4	0.9300
Cu1—Cl1^i^	2.3961 (10)	C5—H5	0.9300
Cu1—Cl1	2.4192 (10)	C8—C9	1.494 (7)
Cu1—Cl2	2.4263 (10)	C8—H8A	0.9700
Cl1—Cu1^ii^	2.3961 (9)	C8—H8B	0.9700
Cl2—Cu1^iii^	2.4263 (10)	O1—Cu1^iii^	1.9199 (4)
N3—C1	1.313 (4)	O1—Cu1^i^	1.9199 (4)
N3—C2	1.394 (5)	O1—Cu1^ii^	1.9199 (4)
C1—N2	1.345 (5)	C9—C10	1.328 (6)
C1—H1	0.9300	C9—N1	1.358 (6)
C2—C3	1.386 (6)	C10—C11	1.480 (8)
C2—C7	1.407 (5)	C10—H10	0.9300
C7—N2	1.395 (6)	N1—C13	1.293 (9)
C7—C6	1.409 (6)	C12—C13	1.301 (12)
N2—C8	1.474 (5)	C12—C11	1.362 (10)
C6—C5	1.337 (8)	C12—H12	0.9300
C6—H6	0.9300	C11—H11	0.9300
C3—C4	1.382 (6)	C13—H13	0.9300
C3—H3	0.9300		
			
O1—Cu1—N3	179.14 (9)	C3—C4—H4	119.7
O1—Cu1—Cl1^i^	85.68 (3)	C5—C4—H4	119.7
N3—Cu1—Cl1^i^	95.10 (9)	C6—C5—C4	122.3 (5)
O1—Cu1—Cl1	85.03 (3)	C6—C5—H5	118.8
N3—Cu1—Cl1	94.25 (9)	C4—C5—H5	118.8
Cl1^i^—Cu1—Cl1	120.483 (17)	N2—C8—C9	113.9 (4)
O1—Cu1—Cl2	83.56 (2)	N2—C8—H8A	108.8
N3—Cu1—Cl2	96.40 (9)	C9—C8—H8A	108.8
Cl1^i^—Cu1—Cl2	117.17 (3)	N2—C8—H8B	108.8
Cl1—Cu1—Cl2	119.88 (3)	C9—C8—H8B	108.8
Cu1^ii^—Cl1—Cu1	80.69 (3)	H8A—C8—H8B	107.7
Cu1—Cl2—Cu1^iii^	81.58 (4)	Cu1^iii^—O1—Cu1^i^	108.564 (12)
C1—N3—C2	105.8 (3)	Cu1^iii^—O1—Cu1	111.30 (2)
C1—N3—Cu1	128.2 (3)	Cu1^i^—O1—Cu1	108.564 (12)
C2—N3—Cu1	126.0 (2)	Cu1^iii^—O1—Cu1^ii^	108.564 (12)
N3—C1—N2	113.1 (4)	Cu1^i^—O1—Cu1^ii^	111.30 (2)
N3—C1—H1	123.5	Cu1—O1—Cu1^ii^	108.564 (12)
N2—C1—H1	123.5	C10—C9—N1	123.5 (5)
C3—C2—N3	131.4 (3)	C10—C9—C8	122.7 (5)
C3—C2—C7	119.6 (4)	N1—C9—C8	113.8 (5)
N3—C2—C7	108.9 (4)	C9—C10—C11	118.1 (6)
N2—C7—C2	104.9 (4)	C9—C10—H10	121.0
N2—C7—C6	134.1 (4)	C11—C10—H10	121.0
C2—C7—C6	121.0 (5)	C13—N1—C9	117.3 (7)
C1—N2—C7	107.4 (3)	C13—C12—C11	123.7 (8)
C1—N2—C8	127.5 (4)	C13—C12—H12	118.1
C7—N2—C8	125.1 (4)	C11—C12—H12	118.1
C5—C6—C7	117.8 (4)	C12—C11—C10	113.3 (7)
C5—C6—H6	121.1	C12—C11—H11	123.3
C7—C6—H6	121.1	C10—C11—H11	123.3
C4—C3—C2	118.6 (4)	N1—C13—C12	123.9 (8)
C4—C3—H3	120.7	N1—C13—H13	118.0
C2—C3—H3	120.7	C12—C13—H13	118.0
C3—C4—C5	120.6 (5)		
			
O1—Cu1—Cl1—Cu1^ii^	−1.10 (2)	N2—C7—C6—C5	−178.9 (5)
N3—Cu1—Cl1—Cu1^ii^	178.42 (9)	C2—C7—C6—C5	3.0 (7)
Cl1^i^—Cu1—Cl1—Cu1^ii^	−83.12 (4)	N3—C2—C3—C4	−178.9 (4)
Cl2—Cu1—Cl1—Cu1^ii^	78.54 (4)	C7—C2—C3—C4	1.4 (7)
O1—Cu1—Cl2—Cu1^iii^	0.0	C2—C3—C4—C5	−0.2 (8)
N3—Cu1—Cl2—Cu1^iii^	−179.13 (9)	C7—C6—C5—C4	−1.8 (9)
Cl1^i^—Cu1—Cl2—Cu1^iii^	81.77 (3)	C3—C4—C5—C6	0.5 (9)
Cl1—Cu1—Cl2—Cu1^iii^	−80.48 (3)	C1—N2—C8—C9	−48.8 (6)
O1—Cu1—N3—C1	150 (6)	C7—N2—C8—C9	135.3 (4)
Cl1^i^—Cu1—N3—C1	−4.5 (3)	N3—Cu1—O1—Cu1^iii^	88 (6)
Cl1—Cu1—N3—C1	116.6 (3)	Cl1^i^—Cu1—O1—Cu1^iii^	−117.99 (3)
Cl2—Cu1—N3—C1	−122.7 (3)	Cl1—Cu1—O1—Cu1^iii^	120.87 (3)
O1—Cu1—N3—C2	−31 (6)	Cl2—Cu1—O1—Cu1^iii^	0.0
Cl1^i^—Cu1—N3—C2	174.8 (3)	N3—Cu1—O1—Cu1^i^	−153 (6)
Cl1—Cu1—N3—C2	−64.1 (3)	Cl1^i^—Cu1—O1—Cu1^i^	1.44 (3)
Cl2—Cu1—N3—C2	56.7 (3)	Cl1—Cu1—O1—Cu1^i^	−119.70 (3)
C2—N3—C1—N2	−0.7 (4)	Cl2—Cu1—O1—Cu1^i^	119.434 (8)
Cu1—N3—C1—N2	178.8 (2)	N3—Cu1—O1—Cu1^ii^	−32 (6)
C1—N3—C2—C3	−178.6 (4)	Cl1^i^—Cu1—O1—Cu1^ii^	122.58 (3)
Cu1—N3—C2—C3	2.0 (6)	Cl1—Cu1—O1—Cu1^ii^	1.43 (3)
C1—N3—C2—C7	1.2 (4)	Cl2—Cu1—O1—Cu1^ii^	−119.434 (8)
Cu1—N3—C2—C7	−178.3 (2)	N2—C8—C9—C10	−102.8 (6)
C3—C2—C7—N2	178.6 (4)	N2—C8—C9—N1	78.9 (5)
N3—C2—C7—N2	−1.2 (4)	N1—C9—C10—C11	1.4 (7)
C3—C2—C7—C6	−2.8 (6)	C8—C9—C10—C11	−176.8 (4)
N3—C2—C7—C6	177.4 (4)	C10—C9—N1—C13	−1.6 (7)
N3—C1—N2—C7	−0.1 (4)	C8—C9—N1—C13	176.8 (5)
N3—C1—N2—C8	−176.6 (4)	C13—C12—C11—C10	−2.7 (10)
C2—C7—N2—C1	0.8 (4)	C9—C10—C11—C12	0.6 (8)
C6—C7—N2—C1	−177.6 (5)	C9—N1—C13—C12	−0.5 (10)
C2—C7—N2—C8	177.4 (4)	C11—C12—C13—N1	2.8 (13)
C6—C7—N2—C8	−1.0 (8)		

Symmetry codes: (i) −*y*+1, *x*, −*z*; (ii) *y*, −*x*+1, −*z*; (iii) −*x*+1, −*y*+1, *z*.
